# The prognosis and clinicopathological features of different distant metastases patterns in renal cell carcinoma: analysis based on the SEER database

**DOI:** 10.1038/s41598-021-97365-6

**Published:** 2021-09-08

**Authors:** Haibin Wei, Jia Miao, Jianxin Cui, Wei Zheng, Xinpeng Chen, Qi Zhang, Feng Liu, Zujie Mao, Songlin Qiu, Dahong Zhang

**Affiliations:** 1 Department of Urology, Zhejiang Provincial People’s Hospital, People’s Hospital of Hangzhou Medical College, No. 158, Shangtang Road, Xiacheng District, Hangzhou, 310014 Zhejiang China; 2 Department of Urology, Taizhou First People’s Hospital, No. 218, Hengjie Road, Huangyan District, Taizhou, 318020 Zhejiang China; 3 Taizhou Hospital, 150 Ximen Street, Linhai, 317000 Zhejiang Province China

**Keywords:** Cancer of unknown primary, Cancer prevention, Urological cancer

## Abstract

Existing data on the prognosis and clinicopathological features of patients with metastatic renal cell carcinoma (mRCC) are limited. This study aims to investigate the prognostic value and clinicopathological features of different metastatic sites in patients with mRCC. A dataset from the National Cancer Institute’s Surveillance, Epidemiology, and End Results (SEER) database consisting of 18 registries (1973–2015) was selected for a retrospective mRCC cohort study. Information was included on the metastatic sites in lung, bone, liver, and brain. Kaplan–Meier analysis was applied to compare the survival distribution. Univariate and multivariate Cox regression models were used to analyze survival outcomes. From the SEER database, a total of 10,410 patients with primary mRCC from 2010 to 2015 were enrolled in this cohort study. Analysis indicated that 54.9%, 37.7%, 19.5%, and 10.4% of patients were found to have lung, bone, liver, and brain metastasis, respectively. There was a significantly higher risk for sarcomatoid RCC patients to develop liver metastasis as compared to patients with clear cell RCC. The median survival for patients with lung, bone, liver, or brain metastasis was 7 months, 7 months, 4 months, and 5 months, respectively. Various clinicopathological features and prognostic values are associated with different metastatic sites. Understanding these differences may enable targeted pre-treatment assessment of primary mRCC and personalized curative intervention for patients.

## Introduction

With the increase in axial cross-sectional imaging, the incidence of kidney cancer has continued to rise over the past three decades^1,2^. Kidney and renal pelvis cancer are the third most diagnosed genitourinary malignancies second only to prostate and bladder cancer, and the estimated number of newly diagnosed cases and deaths in the United States were 73,750 and 14,830, respectively, according to Cancer statistics in 2020^3^. Approximately 80–85% of all primary kidney neoplasms, specifically those which originate from the renal cortex, are clear cell renal cell carcinoma (RCC)^4,5^. In this population, 12–16% of patients will be affected by distant metastasis at diagnosis^3,5^.

In recent years, due to the clinical application of novel vascular endothelial growth factor receptor-tyrosine kinase inhibitors (VEGFR-TKIs) and multiple immune checkpoint inhibitors, there has been significant progress in the treatment of mRCC^6^. Immune-related adverse events remain an area of concern that requires improvement, and choosing the appropriate treatment for different metastatic patterns is becoming complex and challenging^7^. Therefore, knowledge of prognosis and clinicopathological features will assist in arriving at the most optimal clinical decisions for each individual patient.

Since 2010, the Surveillance, Epidemiology, and End Results (SEER) data has been providing metastatic patterns for cancers, including lung, bone, liver, and brain^8^. The lung is the most common site for the occurrence of metastatic disease, and lung metastasis have been reported in 45% of patients with metastatic renal cell carcinoma (mRCC)^9–11^. Bone metastasis from RCC is rare, occurring in only 3.29% of patients at initial diagnosis, but accounts for one-third of patients with mRCC^12–14^. Patients with liver metastasis, which is present in 23.6% of cases of newly diagnosed mRCC, have dismal survival outcomes^15,16^. Brain metastasis will develop in 2.4% of non-metastatic RCC patients, even though the incidence of brain metastasis at diagnosis is 6.5%^13,17^. Due to limited sample size, the incidence rates of metastasis to the above sites may not be sufficiently estimated, and some reports on distant metastasis from RCC are simply case reports. Furthermore, few studies have focused on the distribution and overall survival (OS) of patients with mRCC, and clinicopathological features have been less involved.

Based on the lack of knowledge of the influence of clinicopathological features on disease characteristics, we examined the association between clinicopathology and the distribution of metastatic sites in patients with RCC. On the basis of the previous work of Chandrasekar and Abdel-Rahman, we provide a nomogram prediction of prognosis, metastatic number, and clinicopathological distribution of metastatic sites using a larger modern SEER dataset^10,18^. The objective of this study is to provide guidance regarding the prognosis and clinicopathological features of patients with mRCC.

## Materials and methods

### Database

After the ‘Surveillance, Epidemiology, and End Results Program Data Use Agreement’ was signed in accordance with the requirements for using the SEER database, we obtained permission to access. The datasets analyzed during the current study are available in the SEER database, https://seer.cancer.gov/, and SEER*stat 8.3.6 software was used to extract the data by available code. The 18 population-based cancer registries in the SEER database were selected for this retrospective study. There were 10,410 patients with microscopically confirmed diagnosis of mRCC who were included from 2010 to 2015, because metastatic information regarding liver, lung, bone, and brain was collected after 2010. Other inclusion criteria were as follows: (1) all patients were at stage IV using the 7th edition of the Derived American Joint Committee on Cancer (AJCC) staging system; (2) active follow-up was being conducted for all patients, and age at diagnosis was confirmed; (3) the included patients had specific metastatic details regarding their bone, brain, liver, and lung. Exclusion criteria included tumor behavior that was benign and/or borderline, unknown age, and incomplete survival months.

### Outcome variables

The variables included in the analysis were diagnosis, age, race, gender, Fuhrman grade, tumor, node and metastasis (TNM) classification system (AJCC, 7th edition, 2010), pathological type, insurance status, marital status, metastatic sites, and survival months.

There are five categories of Fuhrman grade: well differentiated (Grade I), moderately differentiated (Grade II), poorly differentiated (Grade III), undifferentiated (Grade IV), and unknown.

We classified race into four groups: “White”, “Black”, “Other,” and “Unknown”.

Based on the International Classification of Diseases for Oncology, 3rd Edition (ICD-O-3) morphology codes, we identified 5 of the highest frequency RCC histological types: clear cell RCC, papillary RCC, chromophobe RCC, sarcomatoid RCC, and collecting duct RCC.

As for insurance status, we reclassified patients into “Insured groups” and “Uninsured groups”. Cases in “Any Medicaid,” “Insured,” and “Insured/No specifics” groups were collapsed into one group named “Insured groups”.

Marital status was defined as married or unmarried. Patients in “Single”, “Separated/divorced”, and “Widowed” were clustered together in the “Unmarried group”. Because of the confusion of the “Unmarried or domestic partner” group, we did not include it in the analysis. The resulting data on survival status, survival time, and cause of death were extricated from the database.

### Statistical analysis

Descriptive statistics were utilized to summarize the patients’ demographic and tumor characteristics. The chi-square test was used to compare the categorical variables, and continuous variables was compared with Student’s *t*-test. Univariate and multivariable logistic regression analyses were implemented to determine if there were any statistical relationships between each independent variable and survival. Only the variables with significance in the univariate analysis can be considered in the multivariate analysis. The hazard ratio (HR) and 95% confidence interval (CI) were utilized to assess the independent risk factors for mRCC in the Cox proportional hazards regression model. All statistical tests were two-sided, and P < 0.05 was regarded as significant. The above analyses were processed using the SPSS 25.0 software package (IBM Corporation, Armonk, NY, USA).

### Ethics approval and consent to participate

All procedures performed in studies involving human participants were in accordance with the 1964 Helsinki declaration and its later amendments or comparable ethical standards. We signed the ‘Surveillance, Epidemiology, and End Results Program Data Use Agreement’ in accordance with the requirement of using SEER database. Approval was waived by the local ethics committee, as SEER data is publicly available and de-identified.

## Results

### Patient characteristics

Overall, there were 10,410 patients who met the screening criteria between 2010 and 2015 based on the SEER database, of which 7161 (68.8%) were male, and 3249 (31.2%) were female. The median age of all patients was 64 years old. As for TNM stage, 1831 (17.6%) patients were in T4 stage, and 2301 (30.7%) were in N1 stage. There were 5713 (54.9%) patients with lung metastasis, 3920 (37.7%) patients with bone metastasis, 2034 (19.5%) patients with liver metastasis, and 1079 (10.4%) patients with brain metastasis. For patients with only one site, 50.6% (5268/10,410) were categorized in RCC stage IV. The detailed clinical features of the mRCC patients are displayed in Table 1.

**Table 1:** Clinical features and metastasis sites for RCC.

	Lung metastasis (%)			Bone metastasis (%)			Liver metastasis (%)			Brain metastasis (%)		
	Yes	No	P	Yes	No	P Value	Yes	No	P Value	Yes	No	P Value
Age at diagnosis	63.7 ± 11.7	65.0 ± 12.2	< 0.001	64.3 ± 11.9	64.2 ± 12.0	0.938	64.0 ± 12.2	64.3 ± 11.9	0.621	61.8 ± 10.3	64.5 ± 12.1	< 0.001
Race			< 0.001			0.044			< 0.001			< 0.001
White	4690 (54.8)	3861 (45.2)		3257 (38.1)	5294 (61.9)		1598 (18.7)	6953 (81.3)		924 (10.8)	7627 (89.2)	
Black	554 (50.4)	546 (49.6)		402 (36.5)	698 (63.5)		292 (26.5)	808 (73.5)		72 (6.5)	1028 (93.5)	
Other	455 (62.2)	276 (37.8)		256 (35.0)	475 (65.0)		139 (19.0)	592 (81.0)		82 (11.2)	649 (88.8)	
Unknown	14 (50.0)	14 (50.0)		5 (17.9)	23 (82.1)		5 (17.9)	23 (82.1)		1 (3.6)	27 (96.4)	
Gender			0.002			0.102			< 0.001			0.665
Male	4002 (55.9)	3159 (44.1)		2734 (38.2)	4427 (61.8)		1321 (18.4)	5840 (81.6)		736 (10.3)	6425 (89.7)	
Female	1711 (52.7)	1538 (47.3)		1186 (36.5)	2063 (63.5)		713 (21.9)	2536 (78.1)		343 (10.6)	2906 (89.4)	
Grade			0.100			< 0.001			< 0.001			< 0.001
Well	91 (51.7)	85 (48.3)		63 (35.8)	113 (64.2)		25 (14.2)	151 (85.8)		16 (9.1)	160 (90.9)	
Moderately	496 (51.1)	474 (48.9)		297 (30.6)	673 (69.4)		118 (12.2)	852 (87.8)		98 (10.1)	872 (89.9)	
Poorly	1185 (54.5)	989 (45.5)		680 (31.3)	1494 (68.7)		341 (15.7)	1833 (84.3)		180 (8.3)	1994 (91.7)	
Undifferentiated	902 (55.7)	718 (44.3)		415 (25.6)	1205 (74.4)		243 (15.0)	1377 (85.0)		129 (8.0)	1491 (92.0)	
Unknown	3039 (55.6)	2431 (44.4)		2465 (45.1)	3005 (54.9)		1307 (23.9)	4163 (76.1)		656 (12.0)	4814 (88.0)	
Histology			< 0.001			0.042			0.001			< 0.001
Clear cell RCC	2615 (57.0)	1970 (43.0)		1625 (35.4)	2960 (64.6)		671 (14.6)	3914 (85.4)		503 (11.0)	4082 (89.0)	
Papillary RCC	216 (41.6)	303 (58.4)		157 (30.3)	362 (69.7)		86 (16.6)	433 (83.4)		30 (5.8)	489 (94.2)	
Chromophobe RCC	29 (34.5)	55 (65.5)		29 (34.5)	55 (65.5)		16 (19.0)	68 (81.0)		6 (7.1)	78 (92.9)	
Sarcomatoid RCC	311 (57.1)	234 (42.9)		210 (38.5)	335 (61.5)		117 (21.5)	428 (78.5)		46 (8.4)	499 (91.6)	
Collecting duct RCC	42 (63.6)	24 (36.4)		28 (42.4)	38 (57.6)		13 (19.7)	53 (80.3)		2 (3.0)	64 (97.0)	
T stage			< 0.001			< 0.001			< 0.001			< 0.001
T1	732 (42.9)	974 (57.1)		927 (54.3)	779 (45.7)		265 (15.5)	1441 (84.5)		165 (9.7)	1541 (90.3)	
T2	994 (64.1)	556 (35.9)		619 (39.9)	931 (60.1)		279 (18.0)	1271 (82.0)		259 (16.7)	1291 (83.3)	
T3	2143 (64.4)	1186 (35.6)		1059 (31.8)	2270 (68.2)		579 (17.4)	2750 (82.6)		311 (9.3)	3018 (90.7)	
T4	815 (44.5)	1016 (55.5)		392 (21.4)	1439 (78.6)		439 (24.0)	1392 (76.0)		121 (6.6)	1710 (93.4)	
Unknown	988 (52.6)	890 (47.4)		864 (46.0)	1014 (54.0)		452 (24.1)	1426 (75.9)		217 (11.6)	1661 (88.4)	
N stage			< 0.001			< 0.001			< 0.001			0.026
N0	3115 (53.1)	2755 (46.9)		2169 (37.0)	3701 (63.0)		931 (15.9)	4939 (84.1)		639 (10.9)	5231 (89.1)	
N1	1869 (58.4)	1332 (41.6)		1179 (36.8)	2022 (63.2)		797 (24.9)	2404 (75.1)		293 (9.2)	2908 (90.8)	
Unknown	729 (54.4)	610 (45.6)		572 (42.7)	767 (57.3)		306 (22.9)	1033 (77.1)		147 (11.0)	1192 (89.0)	
Insurance			< 0.001			0.384			0.633			0.002
Insured	5414 (55.2)	4391 (44.8)		3696 (37.7)	6109 (62.3)		1909 (19.5)	7896 (80.5)		1012 (10.3)	8793 (89.7)	
Uninsured	237 (57.5)	175 (42.5)		160 (38.8)	252 (61.2)		88 (21.4)	324 (78.6)		58 (14.1)	354 (85.9)	
Unknown	62 (32.1)	131 (67.9)		64 (33.2)	129 (66.8)		37 (19.2)	156 (80.8)		9 (4.7)	184 (95.3)	
Married status			< 0.001			0.079			0.025			0.013
Married	3382 (56.3)	2628 (43.7)		2238 (37.2)	3772 (62.8)		1125 (18.7)	4885 (81.3)		628 (10.4)	5382 (89.6)	
Unmarried	2095 (53.8)	1801 (46.2)		1511 (38.8)	2385 (61.2)		814 (20.9)	3082 (79.1)		418 (10.7)	3478 (89.3)	
Unknown	223 (46.2)	260 (53.8)		165 (34.2)	318 (65.8)		90 (18.6)	393 (81.4)		31 (6.4)	452 (93.6)	

*RCC* renal cell carcinoma.

### Lung metastasis

The lung is the most common site for synchronous metastasis in mRCC patients among the cohort with metastatic disease. The mean age of patients without lung metastasis was 1.3 years older than those with lung metastases. White patients had a higher proportion of lung metastasis as compared to patients of other ethnicities. As compared to females, there was a larger percentage of males with lung metastasis. T3 patients had the highest rate of lung metastasis, and T1 patients had the lowest rate when considering patients classified by T stage. For N stage classification, a significantly higher rate of lung metastasis was observed for N1 patients as compared to N0, at 58.4% vs. 53.1%, respectively, P < 0.001. Significantly higher rates were also observed for married and uninsured patients as compared to other types, with P < 0.001 for both. There was no significant difference in lung metastasis when considering the Fuhrman grade.

### Bone metastasis

As for bone metastasis, there was no difference between male and female patients. The same phenomenon occurred for age at diagnosis, insurance status, and marital status. White patients had higher percentages of bone metastasis than black patients and other races. T1 patients accounted for the largest proportion in T stage classification with bone metastasis. Unlike the results of lung metastasis, N0 patients had higher percentages of bone metastasis. For Fuhrman grade, undifferentiated (grade IV) presented the lowest rate.

### Liver metastasis

There are many different outcomes for patients with liver metastasis as compared to bone and lung metastasis. For ethnicity, there was a higher percentage of black patients with liver metastasis as compared to other races. Regardless of gender and marital status, the opposite results were observed for liver metastasis, as there was a greater amount of liver metastasis in females than males, and similarly, more metastasis in unmarried patients than married. Patients in T4 were the most common type in the T stage classification with liver metastasis. In terms of N stage classification, N1 patients exhibited more frequent occurrence of liver metastasis as compared to the other stages. Insurance state and age at diagnosis exhibited no statistically significant difference.

### Brain metastasis

Some features for patients with brain metastasis are similar to those for patients with lung metastasis: age at diagnosis, ethnicity, and T stage classification. There was significant difference between N0 patients and N1 patients with respect to brain metastasis. Unexpectedly, uninsured and unmarried patients exhibited higher percentages than those who were insured and married, both at P < 0.05. Patients with undifferentiated tumors exhibited lower brain metastatic rates than well, moderately, and poorly differentiated tumors. There was no significant difference between males and females.

### Combination of metastases

**Figure 1:**
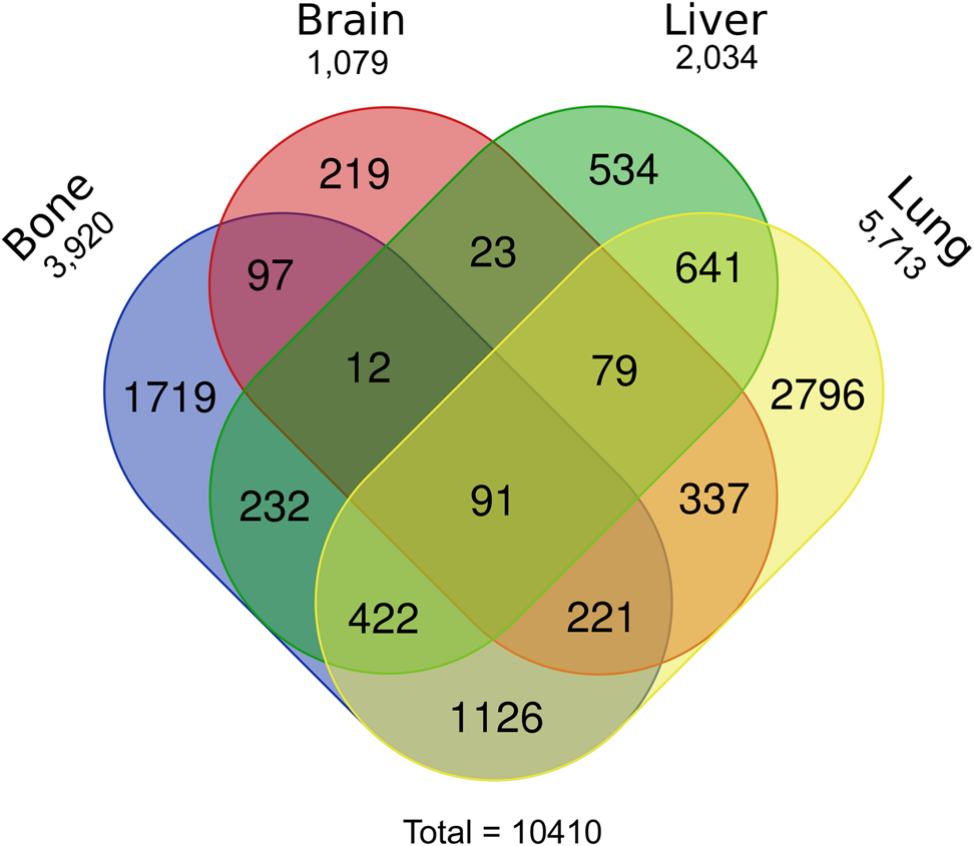
Venn diagram of the distribution of distant metastatic sites. There were four types of metastatic sites in 10,410 patients. Lung metastasis was the most common forms of metastasis).

There were many patients with more than one metastasis. Except for one-site metastasis, 11 combinations of metastases are listed in Table 2. As shown in Fig. 1, a Venn diagram was used to illustrate the distribution of the mRCC patients. For metastasis at two sites, the highest frequency was observed in patients with bone and lung metastasis, at 10.82% (1126/10,410). Only 12 patients had bone and brain and liver metastasis, as this was the least common metastatic combination in mRCC patients. There were 91 mRCC patients with metastasis at all four sites.

**Table 2:** Frequencies of combination metastasis and 3, 5-y OS.

	Number (%)	3-y OS	5-y OS	Median OS (months)
**One site**				
Only Lung	2796 (26.86)	9.05%	1.54%	11
Only Bone	1719 (16.51)	10.99%	2.27%	12
Only Liver	534 (5.13)	6.74%	0.75%	5
Only Brain	219 (2.10)	7.76%	1.37%	9
**Two sites**				
Bone and brain	97 (0.93)	2.06%	0	5
Bone and liver	232 (2.22)	3.45%	0	4
Bone and lung	1126 (10.82)	4.62%	0.44%	6
Brain and liver	23 (0.22)	0	0	3
Brain and lung	337 (3.24)	3.56%	0.30%	6
Liver and lung	641 (6.16)	2.18%	0.16%	4
**Three sites**				
Bone and brain and liver	12 (0.12)	0	0	3
Bone and brain and lung	221 (2.12)	1.81%	0	4
Bone and liver and lung	422 (4.05)	1.81%	0	3
Brain and liver and lung	79 (0.76)	1.27%	0	3
**Four sites**				
Bone and brain and liver and lung	91 (0.87)	0	0	3

OS: overall survival.

### Pathological distribution

**Figure 2:**
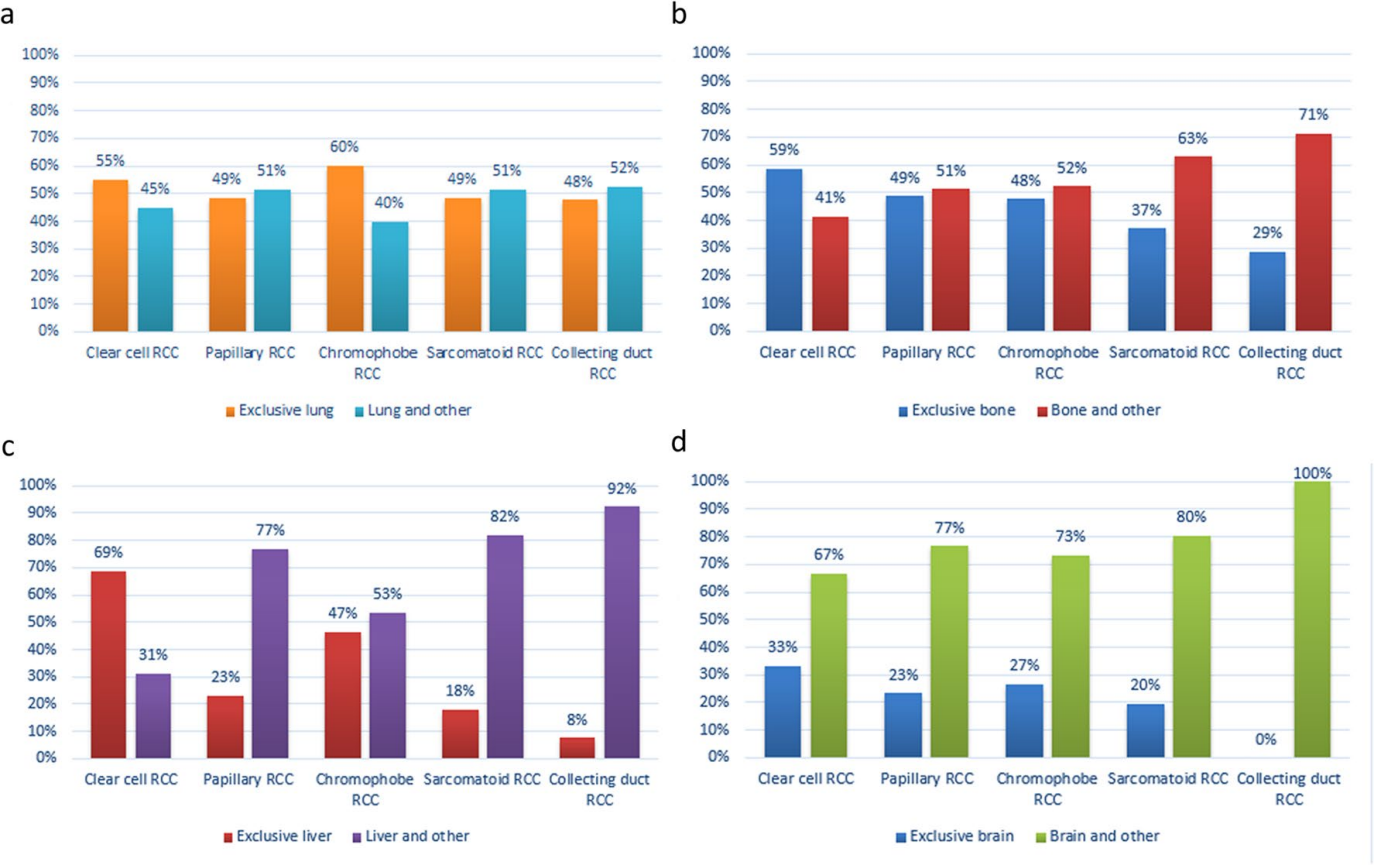
The distribution of metastases in a single versus multiple concomitant sites, stratified by pathology categories, in the four kinds of metastatic sites: lung (**a**), bone (**b**), liver (**c**), and brain (**d**).

Patients were grouped according to the most frequent pathotype in the SEER database, and the difference in pathological distribution of solitary metastasis and synchronous metastases at the time of diagnosis is shown in Fig. 2. In terms of the type of clear cell RCC and chromophobe RCC, the percentage of exclusive lung metastasis was higher than that of synchronous metastases (including the lung). The same phenomenon with clear cell RCC reappeared for bone and liver metastasis. It is interesting that there were differences in liver metastasis between pathological types. Except for clear cell RCC, there were higher percentages for liver plus other metastases than for exclusive liver metastasis (Fig. 2c). Brain and other metastases occurred with much greater frequency than exclusive brain metastasis for all pathological types (Fig. 2d). Univariate survival analysis of distant metastases sites.

Table 3 lists variables including the metastatic site, ethnicity, gender, grade, T stage, N stage, insurance status, and marital status. All variables were regarded as prognostic factors relating to overall survival apart from insurance status. For one-site metastasis out of four metastatic sites, the worst OS was for patients with liver metastasis. Among the cohort, bone metastasis presented the minimum hazard ratio (HR). Black patients exhibited worse prognoses as compared to white patients for OS (P < 0.001). Compared to males, there were worse prognoses for females. An interesting phenomenon was observed for Fuhrman grade, where patients with moderately differentiated grade appeared to have better OS than patients with well differentiated grade (P = 0.003). Similarly, T3 patients seemed to exhibit a survival advantage when compared to T1 patients. As we expected, N1 patients had worse prognoses as compared to N0 patients.

**Table 3:** Univariate survival analysis of patients with four single metastases.

Characteristics	Overall survival	
	HR (95% CI)	p-value
Metastatic site		< 0.001
Lung	1	Ref
Bone	0.969 (0.901–1.042)	0.393
Liver	1.490 (1.341–1.655)	< 0.001
Brain	1.118 (0.948–1.319)	0.185
Race		< 0.001
White	1	Ref
Black	1.151(1.071–1.238)	< 0.001
Other	0.930(0.850–1.018)	0.115
Gender		0.020
Male	1	Ref
Female	1.060(1.009–1.113)	0.020
Grade		< 0.001
Well	1	Ref
Moderate	0.734(0.597–0.903)	0.003
Poorly	1.076(0.886–1.308)	0.460
Undifferentiated	1.358(1.115–1.653)	0.002
Histology		< 0.001
Clear cell RCC	1	Ref
Papillary RCC	1.327 (1.188–1.482)	< 0.001
Chromophobe RCC	0.896 (0.679–1.181)	0.435
Sarcomatoid RCC	2.513 (2.273–2.778)	< 0.001
Collecting duct RCC	2.110 (1.607–2.770)	< 0.001
T stage		< 0.001
T1	1	Ref
T2	1.092 (1.005–1.186)	0.038
T3	0.905 (0.843–0.972)	0.006
T4	1.222 (1.130–1.322)	< 0.001
N stage		< 0.001
N0	1	Ref
N1	1.770 (1.682–1.862)	< 0.001
Insurance status		0.111
Insured	1	Ref
Uninsured	1.096 (0.979–1.228)	0.111
Married status		< 0.001
Married	1	Ref
Unmarried	1.241 (1.184–1.301)	< 0.001

*HR* hazard ratio, *CI* confidence interval, *Ref* reference, *RCC* renal cell carcinoma.

**Figure 3:**
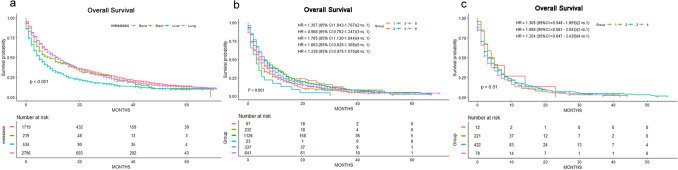
Kaplan–Meier curves and Log-Rank test for OS according to the number of metastasis. (**a**) Only one site metastasis. (**b**) Two sites metastases. 1, Bone and brain metastases; 2, Bone and liver metastases; 3, Bone and lung metastases; 4, Brain and liver metastases; 5, Brain and lung metastases; 6, Liver and lung metastases. (**c**) Three sites metastases. 1, Bone and brain and liver metastases; 2, Bone and brain and lung metastases; 3 Bone and liver and lung metastases; 4, Brain and liver and lung metastases; *HR* hazard ratio).

As for histology, the worst OS was for sarcomatoid RCC patients as compared to RCC that originated from epithelium. There was a significant difference where marital status was concerned, with unmarried patients being prone to worse outcomes. We utilized Kaplan–Meier analysis to create survival curves among the patients with single metastasis and two-site metastasis (Fig. 3a,b). As for the patients with three-site metastasis, the log-rank tests showed that there was no significant difference between them (Fig. 3c).

### Multivariate survival analysis of distant metastases sites

On multivariable Cox regression, ethnicity, gender, and marital status were not independent factors for mRCC (P > 0.05). As for metastatic site, liver metastasis was still the worst prognostic metastasis. An interesting situation arose, where moderately differentiated grades with better OS reappeared upon multivariate survival analysis. Patients with sarcomatoid RCC had worse outcomes as compared to other histological types as well. When the T stage was included in the multivariate survival analysis, T3 patients exhibited higher survival than T1 patients. Regional lymph nodes negative was the positive factor for stage IV patients (Table 4).

**Table 4:** Multivariate survival analysis of patients with four single metastases.

Characteristics	Overall survival	
	HR (95% CI)	p-value
**Metastasis site**		
Lung	1	Ref
Bone	0.896 (0.765–1.050)	0.175
Liver	1.201 (0.932–1.546)	0.156
Brain	1.213 (0.878–1.675)	0.242
**Race**		
White	1	Ref
Black	0.862 (0.661–1.124)	0.273
Other	0.827 (0.649–1.052)	0.124
**Gender**		
Male	1	Ref
Female	1.052 (0.911–1.214)	0.489
**Grade**		
Well	1	Ref
Moderate	0.934 (0.594–1.470)	0.769
Poorly	1.109 (0.709–1.733)	0.651
Undifferentiated	1.335 (0.848–2.102)	0.212
**Histology**		
Clear cell RCC	1	Ref
Papillary RCC	1.509 (1.159–1.964)	0.002
Chromophobe RCC	0.968 (0.567–1.652)	0.905
Sarcomatoid RCC	2.072 (1.662–2.585)	< 0.001
Collecting duct RCC	2.457 (1.429–4.225)	0.001
**T stage**		
T1	1	Ref
T2	1.061 (0.833–1.351)	0.634
T3	1.125 (0.915–0.382)	0.264
T4	1.745 (1.345–2.265)	< 0.001
**N stage**		
N0	1	Ref
N1	1.749 (1.514–2.021)	< 0.001
**Married status**		
Married	1	Ref
Unmarried	1.145 (0.998–1.314)	0.054

*HR* hazard ratio, *CI* confidence interval, *Ref* reference; *RCC* renal cell carcinoma.

### Construction of a prognosis model for distant metastases sites

**Figure 4:**
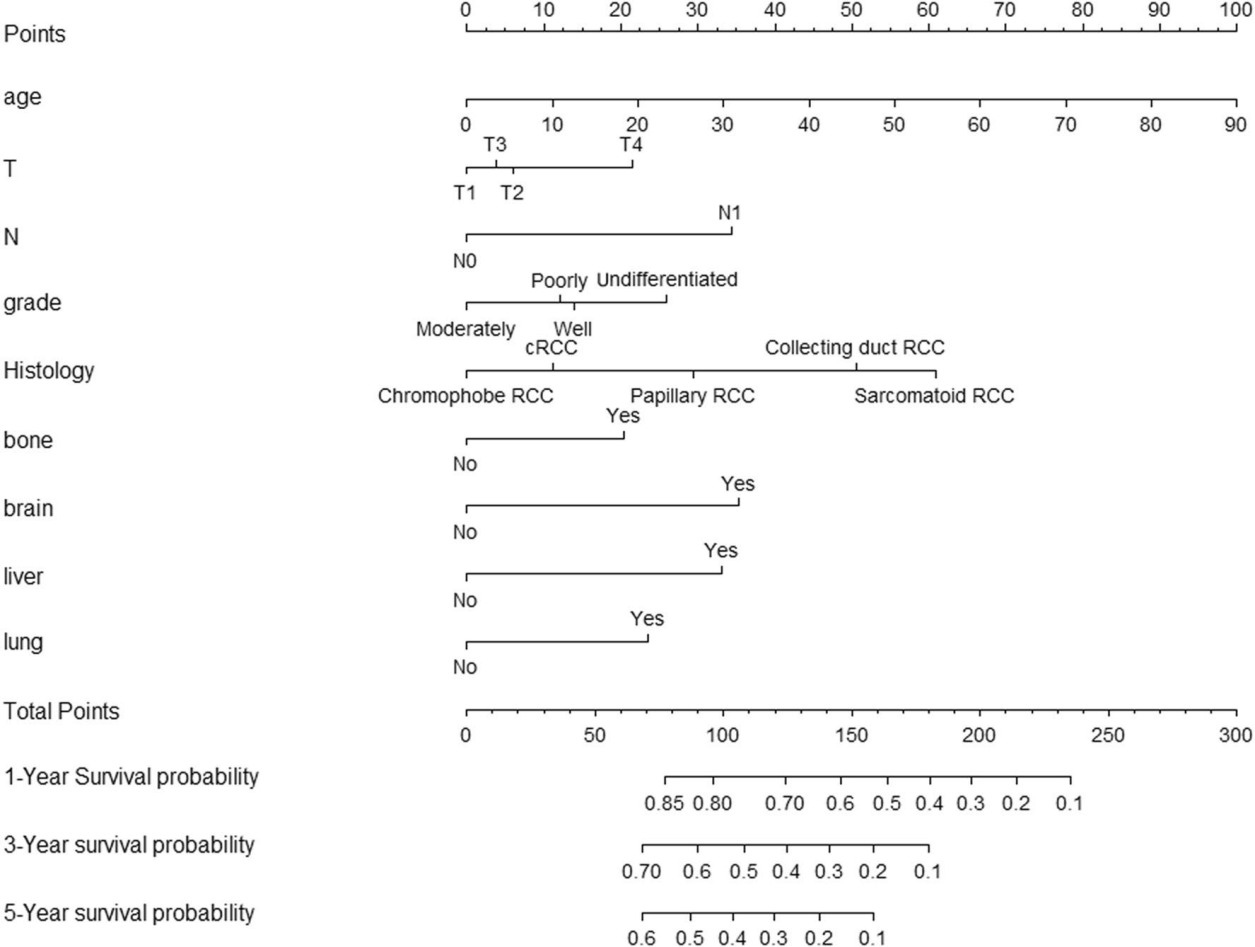
Nomogram for predicting the 1-year, 3-year and 5-year overall survival in patients with primary metastatic renal cell carcinoma. To obtain the predicted survival probability in meters, locate patient values on each axis. Based on the points line to acquire the number of points to add. Sum the points of all variables to determine the total point. A vertical line can be drawed down to the 1-year, 3-year and 5-year overall survival probability).

Meaningful factors were selected for the nomogram model construction that relied on the multivariate survival analysis and clinical availability. The included factors were age, grade, T/N stage, histology, and distant metastases sites. Every factor had an accompanying score that corresponded to the points at the top of the nomogram. For instance, in N stage, 0 points were assigned for N0, and 32 points were assigned for N1. The 1-year, 3-year, and 5-year survival rates were acquired based on the commensurate points. With 160 total points, the 1-year survival rate is 52%, the 3-year survival rate is 20%, and the 5-year survival rate is 10% (Fig. 4).

## Discussion

RCC is one of the deadliest urological malignancies and has a dismal late-stage prognosis, with a 5-year survival rate of only 12% for metastatic disease^19^. SEER breaks the barrier of minor case series and isolated institutional studies and provides a platform for deeply learning about metastatic RCC. In this study, we analyzed RCC with respect to distant sites of metastasis, including bone, brain, liver, and lung, based on the recorded sites in the SEER database from 2010 to 2015. The data from our analysis might provide clinicians with some useful information for each individual patient in terms of diagnosis, prognosis, and other aspects. For example, knowledge of metastatic site distribution may be helpful to design personalized examinations for RCC patients to determine early if there are other merged metastases. By integrating clinical and pathological factors, we establish a comprehensive and practical nomogram to estimate the 1-, 3- and 5-year prognosis for RCC patients.

Within the current study, we identified 10,410 individuals with mRCC between years 2010 and 2015. The number of patients enrolled was significantly more than previous studies accessed from the SEER database, which was 6610 for Chandrasekar and colleagues, and 5992 for Abdel-Rahman and colleagues^10,18^. In our study, the rate of metastases to the lung, bone, liver, and brain was 54.9%, 37.7%, 19.5%, and 10.4%, respectively. The metastatic rates for metastases to three out of the four sites mentioned above were similar to those of previous reports, which were 45.2–51.2% for lung metastasis, 17.0–20.3% for liver metastasis, and 8.1–9.8% for brain metastasis^10,13^. The metastatic rate to bone was slightly higher than what was mentioned in previous literature reports, which was 20–33.5% for bone metastasis^10,12^.

Although bone metastasis was initially underestimated, this situation is correcting itself, which could be due to the following causes. (1) More effort has been made to accurately evaluate the status of metastasis to the above sites using appropriate modalities. The NCCN and EAU recommend bone imaging in symptomatic patients or in those with an abnormal alkaline phosphatase (ALP) level^20,21^. In the presence of an elevated ALP or clinical symptoms, the probability of a positive bone scan increases from approximately 5% to 10%^22^. (2) An increasing number of biochemical markers are emerging, and some of them will play a role in the diagnosis of bone metastasis for RCC patients now and in the future. The “vicious cycle” hypothesis is used to describe how RCC cells interact with the bone microenvironment to drive bone destruction and tumor growth^14^. In this process, many biomarkers and signaling pathways play a role, including TGF-β, TGF-α/EGF-R signaling, insulin mRNA binding protein-3 (IMP3), cadherin-11, PTHrP, calcium/CaSR, AKT/integrin-α5 signaling, matriptase, MET, and miRNAs. Klepzig found that the procollagen type 1 amino-terminal propeptide (P1NP) concentration was significantly higher among those with bone metastasis than in those without^23^. This suggests that P1NP may be a significant early predictor for RCC bone metastasis and may play a certain role in the initial diagnosis. (3) Previous research found that in patients with lung or liver metastasis, there is a higher risk of bone metastasis as compared to those without lung or liver metastasis in colorectal cancer and gastric cancer^8,24^. Our study showed a similar phenomenon when examining multiple metastases in RCC. The number of combined bone metastasis was higher than that of exclusive bone metastasis, for sarcomatoid RCC, collecting duct RCC, papillary RCC, and chromophobe RCC. This association is helpful for us to design screening strategy. Once the other metastases occur, bone scanning can help to decrease the rate of bone metastasis. Knowledge of metastatic site distribution may be helpful for clinicians so that they can design personalized examinations for RCC patients.

The data from the current analysis indicates that the highest survival is for patients with chromophobe RCC and clear cell RCC, which is similar to that found by Abdel-Rahman and colleagues^18^. The rate of metastases in a single site was 50.6% versus 49.4% in two or more sites. Compared to exclusive liver metastasis, sarcomatoid RCC, collecting duct RCC, and papillary RCC are more prone to developing multiple metastases. For all clinicopathological types, brain metastasis did not tend to appear alone and were more likely to be associated with other metastases. Our analysis found that metastatic RCC patients have the worse survival when there is an increase in metastasis sites. We therefore guessed that metastatic disease burden was associated with increased sites, and there might be less time for intervention with these patients.

Our subsequent assessment of survival analysis of metastatic disease arrived at results similar to those previously reported^10,18^. For our univariate survival analysis of patients with four single metastasis at the time of diagnosis, the statistically significant parameters were disease-specific factors such as metastatic site, race, gender, grade, histology, T stage, N stage, and marital status. Among the parameters mentioned above, metastatic site plays an important role. When specifically considering the multivariate survival analysis of patients with four single metastasis, the same factors, including metastatic site, grade, histology, T stage and N stage, predicted a worse prognosis for metastasis. In univariate survival analysis, our study showed that unmarried RCC patients experienced worse overall survival as compared to married patients, which was attributed to the possibility that the spouse might provide social support and encourage the patients to seek medical treatment.

The outcomes for RCC patients with metastasis was poor, which were 7 months, 7 months, 4 months, and 5 months for metastasis to the lung, bone, liver, and brain, respectively. The nomogram is a convenient graphical representation of a mathematical model, in which various important factors are combined to predict a future endpoint^25^. By integrating clinical and pathological factors, the nomogram was used to provide visual estimates of the 1-, 3- and 5-year survival rates of patients in the study. To date, several RCC nomograms have been generated for predicting the probability of RCC recurrence and survival^25–27^. The first nomogram was designed in 2001 by Kattan et al.^25^ to calculate the likelihood of recurrence after surgery for RCC patients. Sorbellini et al. published a postoperative prognostic nomogram in 2005 that was designed to predict recurrence for patients with conventional clear cell RCC^27^. Zhang et al. developed a nomogram to predict the overall survival and the disease-specific survival for clear cell RCC treated with nephrectomy^26^. Due to the important effect of metastasis on prognosis, the aim of the current study was to establish a comprehensive and practical nomogram based on distant metastasis sites for predicting the survival rate of RCC patients. Meaningful factors were selected for the nomogram model construction that relied on multivariate survival analysis and clinical availability in the study.

Novel therapeutic options have brought more significant therapeutic benefits to metastatic RCC patients in the last decade, such as multiple multikinase inhibitors and immune checkpoint inhibitors^7^. Unfortunately, the variable incorporation of therapeutic options and clinical risk scores into the trial design, and the lack of head-to-head trials have made it difficult for urologists and oncologists to select first-line treatments for mRCC patients^28^. Our study focuses on the prognostic value and clinicopathological features of different metastatic sites, not on treatment strategies for mRCC patients. The optimization of treatment strategies will be an important part of subsequent research. The difference in the metastasis sites with respect to treatment methods might provide effective reference information for clinical decision-making. For example, although liver metastases systemically diminish immunotherapy efficacy in patients and preclinical models, the combination of liver-directed radiotherapy and immunotherapy could promote systemic antitumor immunity^29^. Additionally, sequencing and a combination of systemic therapy for different metastasis sites will become a heavily researched area.

There are several limitations to our study due to the limited information in the SEER database. First, the metastatic data for the above 4 sites were provided from 2010 to the present, and thus, the follow-up time is not very long. Further analysis was prevented because of potential confounders due to the lack of effective information on systemic treatment regimens or surgery for some metastatic sites, which may bring bias to the prognosis. Second, compared to those patients with synchronous metastasis, there may be larger quantities of metachronous metastasis. Additionally, there was no information in the database on other metastatic sites, such as the ovaries, other urinary and gastrointestinal system sites, and adrenal gland.

Furthermore, the SEER is an observational retrospective database relying on ICD codes for assessment of secondary diagnostic codes, which may be subject to potential coding biases. The retrospective nature of the SEER database may lead to incomplete or even biased information collection. Despite these limitations from SEER, our population represents the largest cohort used for the assessment of different site-specific mRCC. Our data are highly generalizable since they originate from a nationwide sample, and this might provide some useful knowledge that can be used to predict clinical outcomes and guide decisions regarding surgery, surveillance, and adjuvant therapies. The SEER database is currently updating and expanding its database, and it is likely that additional data will soon be available for analysis.

## Conclusion

Heterogeneity exists in the oncological outcomes of mRCC patients with site-specific metastasis. The highest oncologic survival was experienced by patients with bone metastasis, and the lowest survival was for those with brain metastasis among those with single metastasis. Relying on different histological types, there are numerous metastatic features and prognostic values. Knowledge of these differences in metastatic patterns may assist in designing a targeted pre-treatment assessment of renal cell carcinoma and implementing a personalized curative intervention.

## Data Availability

The datasets analyzed during the current study are publicly available for use in accordance with a limited use agreement for SEER research data: Surveillance, Epidemiology, and End Results (SEER) Program (https://seer.cancer.gov) SEER*Stat Database.
